# Microfluidic biosensors for rapid detection of foodborne pathogenic bacteria: recent advances and future perspectives

**DOI:** 10.3389/fchem.2025.1536928

**Published:** 2025-01-29

**Authors:** Jian Zhang, Chuanlong Ma, Yaping Du, Jiangbo Huang, Li Xue

**Affiliations:** ^1^ School of Robot Engineering, Yangtze Normal University, Chongqing, China; ^2^ Department of Building Environment and Energy Engineering, The Hong Kong Polytechnic University, Hong Kong, China; ^3^ Beijing Key Laboratory of Microanalytical Methods and Instrumentation, Key Laboratory of Bioorganic Phosphorus Chemistry and Chemical Biology, Ministry of Education, Department of Chemistry, Tsinghua University, Beijing, China

**Keywords:** microfluidic biosensors, food safety, rapid analysis, foodborne pathogens, lab-on-chip

## Abstract

Rapid detection of foodborne pathogenic bacteria is critical for ensuring food safety and preventing foodborne disease outbreaks. Traditional detection methods, while accurate, are often time-consuming and labor-intensive, making rapid detection technologies a pressing need. Microfluidic biosensors have emerged as a powerful solution, offering high sensitivity, specificity, and rapid analysis with minimal sample volume. In this review, we summarize recent advances in microfluidic biosensor technology, highlighting innovations in detection techniques such as electrochemical and optical microfluidic biosensors. We have also introduced microfluidic components, which are crucial for the implementation of microfluidic biosensors. Based on the current state of this technology development, we finally provide several most important recommendations for future research directions in this emerging research area, which may enable widespread commercialization and adoption in the food industry.

## 1 Introduction

In recent years, food safety has become a focal point of global public concern (Mao and Hao, 2024). According to reports from the World Health Organization (WHO), each year, approximately 10% of the global population suffers from illnesses related to the consumption of contaminated food, with nearly 2 million deaths attributed to foodborne diseases (Cheng et al., 2023; Organization, 2024). Foodborne diseases can lead to intestinal inflammation, diarrhea, chronic kidney diseases, reactive arthritis, blindness, and even death (Pires et al., 2021; Mi et al., 2022). The primary pathogens responsible for foodborne illnesses include viruses, bacteria, fungi, and parasites. Among these pathogens, bacteria are the most common. The most frequently encountered foodborne pathogenic bacteria include *Salmonella*, *Vibrio parahaemolyticus*, *Bacillus cereus*, *Staphylococcus aureus, enterohemorrhagic Escherichia coli*, *Listeria monocytogenes*, *Campylobacter jejuni*, *Shigella*, and *Cronobacter sakazakii* (Shen et al., 2020; Xiao et al., 2022). Indeed, foodborne outbreaks caused by bacterial pathogens occur frequently worldwide. In 2017, a food safety incident involving *Salmonella enteritidis*-contaminated eggs affected multiple European countries, resulting in 196 confirmed cases (Mihalache et al., 2022). In 2020, an outbreak in the United States (US) linked to *Salmonella*-contaminated onions caused 1,127 cases of illness across 48 states of the US (McCormic et al., 2022). According to cost estimates by the United States Department of Agriculture (USDA), bacterial pathogens account for over 95% of foodborne illness cases and fatalities, imposing an economic burden of approximately $17.6 billion annually (United States Department of Agriculture, 2021).

Previous studies have revealed that bacterial contamination of food can occur at any stage of the food supply chain, from production and processing to transportation and retail (Wang et al., 2020). Early screening for foodborne pathogens is a critical measure to reduce the probability of large-scale foodborne outbreaks and ensure food safety (2019, Food Safety Regulatory Research Needs 2030; European Food Safety et al., 2019; Pires et al., 2021). Although there are many available techniques for detection of foodborne pathogens in food supply chain industry, the screening of foodborne pathogens faces several challenges: 1) the complex matrix of food samples often interferes with detection, leading to inaccurate results; 2) pathogens are typically present at low concentrations during routine screening, producing weak signals that are difficult to detect directly; 3) many sensitive detection instruments are bulk and expensive, making them impractical for in-field use in the food supply chains; 4) the automation level of existing detection methods and instruments remains insufficient, hindering their application for on-site rapid screening of foodborne pathogens. Therefore, advancing research on rapid detection technologies for foodborne pathogens and enhancing risk monitoring and early warning systems are of critical importance and hold significant practical value for ensuring food safety.

Traditional methods for detecting foodborne pathogens primarily include culture-based techniques (Kim and Kim, 2020), immunological methods based on enzyme-linked immunosorbent assay (ELISA) (Wu et al., 2019), and molecular biological methods utilizing polymerase chain reaction (PCR) (Lu et al., 2024). While each of these detection technologies has unique advantages for foodborne pathogen screening, there remain several challenges that need to be addressed urgently. Fortunately, with the interdisciplinary integration of fields such as engineering, biology, chemistry, materials science, and physics, a variety of biosensors have emerged for the rapid detection of foodborne pathogens (Zhao et al., 2024; Feucherolles, 2025).

**FIGURE 1:**
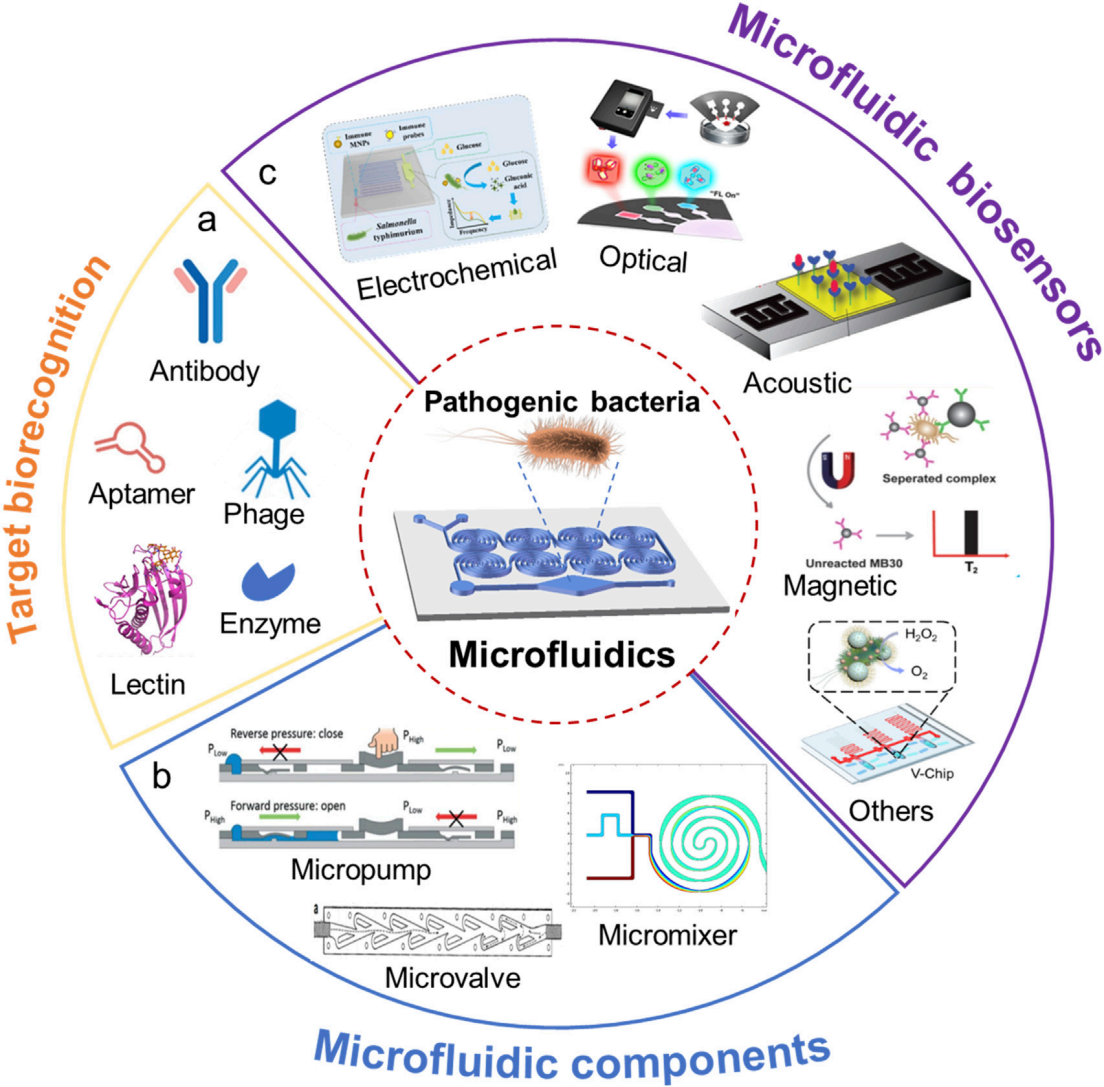
Microfluidic chip for pathogenic bacteria analysis. **(A)** Target biorecognition in biosensors; **(B)** Microfluidic components in a chip; **(C)** Various microfluidic biosensors.

With the rapid advancement of microfluidic technology, the integration of biosensing methods with microfluidic chips has led to the development of a series of novel microfluidic biosensors, enabling on-site detection with “lab-on-a-chip” and “sample-in-answer-out” capabilities (Yafia et al., 2022). Microfluidic biosensors combine microfluidic technology with biorecognition elements, guiding sample liquids through microscale fluidic channels on a chip while employing biorecognition elements to specifically bind with target analytes. As shown in Figure 1A, the target biorecognition elements (such as antibody, enzyme, aptamer, phage, or lectin) can recognize the target (such as cells of pathogenic bacteria, nucleic acid, antigens) from a sample, thereby generating detectable signal changes. The signal changes can be converted and analyzed using various methods such as electrochemical, optical, and mass spectrometry techniques. Because of their special working principle, microfluidic biosensors offer several key advantages, including low sample and reagent consumption, operational flexibility, high integration, and short detection times. Furthermore, they can easily be integrated with technologies such as electrical, magnetic, and optical systems, enabling rapid identification and detection of target analytes. Due to these inherent characteristics, microfluidic biosensors have garnered significant attention and hold great potential for applications in the detection of foodborne pathogens (Bahavarnia et al., 2022; Gao et al., 2022).

Over the past decades, enormous efforts have been dedicated to microfluidic biosensors for rapid detection of foodborne pathogenic bacteria. Many novel and different types of microfluidic biosensors, such as fluorescent biosensors (Jin et al., 2023), and colorimetric biosensors (Xing et al., 2024), have been developed. Although there are already some reviews existing in literature, they generally focus either on working principles and structures of specific microfluidic biosensors (Wu et al., 2024; Zhang et al., 2024), or their applications in different fields (Xing et al., 2022). Considering the very fast development of this emerging field, an up-to-date review is required to comprehensively evaluate the effectiveness of pathogen detection and the distinctive features of the newly developed microfluidic biosensors.

This review aims to summarize recent advances in microfluidic biosensors for rapid detection of foodborne pathogenic bacteria. The specific focus is placed on the newly developed novel microfluidic biosensors classified by the fundamental working principles for food safety monitoring. In the following sections, we first introduce the fundamentals of microfluidic biosensors, including the key components of a microfluidic chip (Figure 1B). Then we examine the different types of microfluidic biosensors for rapid detection of foodborne pathogenic bacteria (Figure 1C). Several recommendations for future research directions and consolidation conclusions are given in the following section of this review. Finally, a general conclusion is provided.

## 2 Fundamentals of microfluidic biosensors

With the growing demand in the field of Point-of-Care Testing (POCT) and the integration of multidisciplinary technologies, biosensors have developed vastly and garnered significant attention from researchers. Meanwhile, microfluidic chips, with their advantages of lab-on-chip control, micro-scale analysis, and small size, have become a powerful tool when integrated with biosensors, driving the rapid advancement of microfluidic biosensors. In order to better understand the fundamentals of microfluidic biosensors, the following sections will introduce the principle of the biosensor, microfluidic biosensors, and the functional components of microfluidic chips.

### 2.1 Biosensors

The principle of biosensors was defined by the International Union of Pure and Applied Chemistry (IUPAC) as follows: “A biosensor is a self-contained integrated device which is capable of providing specific quantitative or semi-quantitative analytical information using a biorecognition element (biochemical receptor) which is in direct spatial contact with a transducer element” (Thévenot et al., 2001). In other words, Biosensor is an analytical device which changes a biological response into a measurable signal. The main components of a biosensor are 1) target biorecognition elements (such as antibody, enzyme, aptamer, phage, or lectin) which recognizes the target (such as cells of pathogenic bacteria, nucleic acid, antigens) from sample, 2) a chemical or physical transducer (such as Microelectrodes, Piezo Quartz Crystals, Field Effect Transistors, Fiber Optics, Surface Plasmon Resonance and Thermistors) that converts the biological response into a readable signal, and 3) a reader for signal readout. According to the different transducers, biosensors can be classified into electrochemical biosensors, optical biosensors, acoustic biosensors, magnetic biosensors and thermal biosensors, etc (Xue et al., 2023).

### 2.2 Microfluidic biosensors

Microfluidic biosensor is a kind of newly emerging biosensor which integrate a series of functions, including sample transfer, target capture, reagent mixing and separation, biochemical reactions, and detection (signal output), into a chip-based system (Wang et al., 2022). Their fundamental principle involves incorporating biosensing detection methods into microfluidic chip platforms. When designing and fabricating microfluidic chips, both the structural and functional design of the chip and the selection of materials play crucial roles. Various materials, such as silicon, glass, quartz, polymethyl methacrylate (PMMA), hydrogels, polydimethylsiloxane (PDMS), paper, fabric, thread, and wood, can be used for constructing two-dimensional (2D) and three-dimensional (3D) microfluidic chips (Ren et al., 2013; Açıkgöz et al., 2023). Additionally, multiplex assay strips made of multicore fibers (MCFs) have been utilized for microbial analysis. When designing microfluidic biosensors, the properties of the target analytes must be considered. The structural design of the chip should integrate processes for recognizing, separating, and detecting the target analytes. This involves selecting appropriate chip fabrication materials, designing chip structures, and choosing compatible signal detection instruments. Ultimately, this approach facilitates the development of microfluidic biosensors for rapid, highly sensitive, and high-throughput detection of foodborne pathogens. Therefore, the microfluidic chip is the key component for a typical microfluidic biosensor.

### 2.3 Microfluidic chip

Microfluidic chip technology, also called Micro Total Analysis System (μTAS) or Lab on a chip (LOC), was first introduced by Manz et al. in 1990s, referring to the technology of finely controlling and manipulating fluids by processing extremely small amounts of fluids through microscale (several microns to hundreds of microns) of fluid channels (Manz et al., 1990; Reyes et al., 2002). Microfluidic chip miniaturized systems, featured with many advantages including 1) precise control of liquids flowing usually under laminar regime, 2) small amount of consumption of reagents and samples, 3) short reaction times, 4) highly parallel and multiplexed analysis, 5) little or less power to operate, 6) portable size, and potentially having low cost of production compared to bulkier analytical instruments (Pattanayak et al., 2021). Over 30 years of development, LOC have demonstrated their potential and benefits for many applications, including detection of foodborne biohazards, point-of-care diagnostics, genomic and proteomic research, analytical chemistry, and environmental monitoring, etc (Manessis et al., 2022; Ao et al., 2024).

An ideal microfluidic chip should be capable of performing all steps of the analytical process, including sample loading, preprocessing, separation, dilution, mixing, reaction, and detection. To achieve these functionalities, it is often necessary to develop and integrate microfluidic components such as 1) micropumps, 2) microvalves, and 3) micromixers on the chip to carry out various experimental operations. With the rapid advancement of microfluidic technology, microfluidic devices are receiving increasing attention. Below we will describe the key components in a typical microfluidic chip.

#### 2.3.1 Micropumps

**FIGURE 2:**
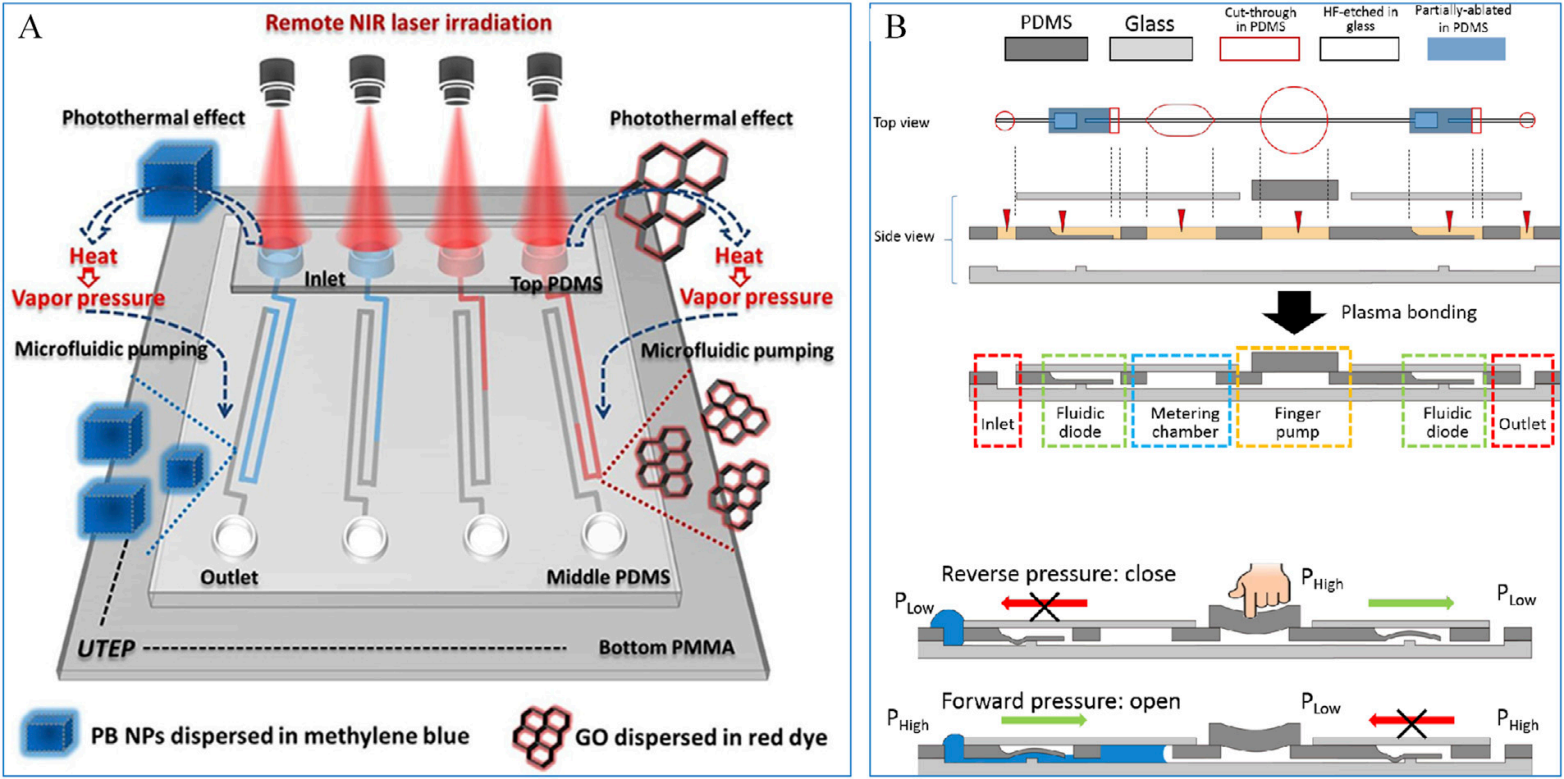
**(A)** A photothermal micropump based on Prussian blue and Graphene Oxide mediation (Fu et al., 2020); **(B)** A micropump based on finger pressure (Xu et al., 2015).

The pumping mechanisms in microfluidic chips typically rely on external standalone pumps, such as constant-pressure pumps, syringe pumps, and peristaltic pumps, to provide fluidic power. However, these external components make the entire microfluidic system bulky, expensive, and operationally complex. To address these challenges, simpler pumping systems have been developed and either integrated with microfluidic chips or directly embedded within them. This advancement has significantly broadened the application of microfluidic chips in point-of-care testing (POCT). For instance, Safavieh et al. (2015) designed a capillary pump using capillary action, where precise control over the pump’s pressure and flow rate was achieved by adjusting the size and spacing of microcolumns. As shown in Figure 2A, Fu et al. (2020) developed a photothermal micro pump driven by the photothermal effect mediated by nanomaterials. Using Prussian Blue (PB) and Graphene Oxide (GO) as photothermal agents, laser irradiation at a wavelength of 808 nm heats the nanomaterial solution to generate steam, which provides the driving force for fluid transport. Xu et al. (2015) created a modular finger-actuated micropump leveraging the elasticity of polydimethylsiloxane (PDMS) membranes. By pressing and releasing the membrane with a finger, the fluid can be directionally transported, enabling multiple parallel operations. As shown in Figure 2B, by producing pressure difference to make fluidic diode enable one-way liquid flow in a finger-driven fluidic circuit. Liquid flow is only permitted from the inlet through the diode under forward pressure. Experimental results demonstrated that this finger-actuated micropump achieved accuracy comparable to that of a pipette.

#### 2.3.2 Microvalves

**FIGURE 3:**
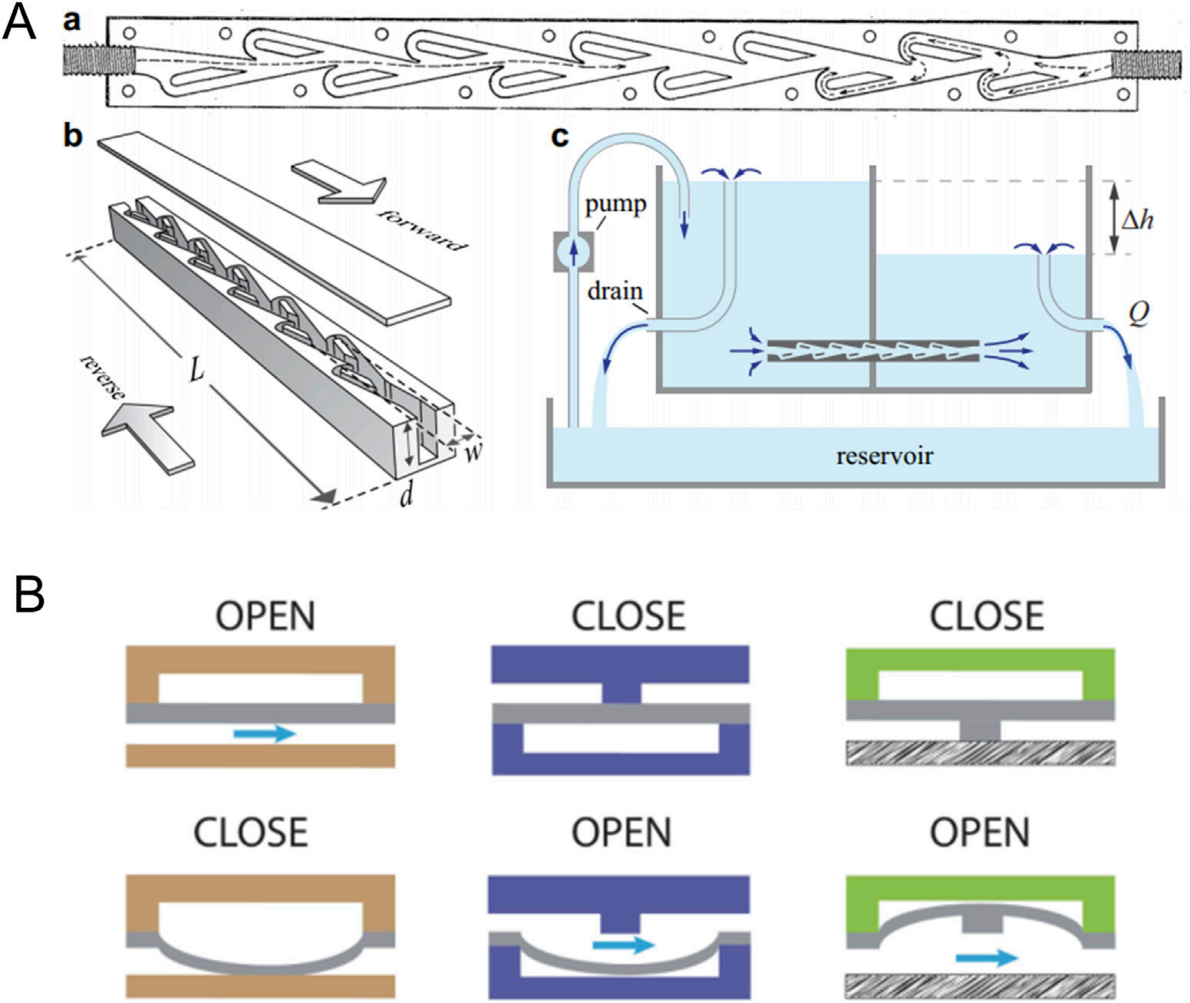
**(A)** A one-way microvalve based on Tesla structure (Nguyen et al., 2021); **(B)** An elastomeric microvalve.

Microvalves are another critical component of microfluidic chips, primarily responsible for channel opening and closing, flow regulation, and reagent sealing. Integrating microvalves into microfluidic chips simplifies the system structure, enhances user convenience, and further strengthens the advantages of microfluidic chips in point-of-care testing (POCT). However, developing microvalves for microfluidic chips poses significant challenges, as it often requires constructing movable structures at millimeter or even micrometer scales. To improve the performance of microvalves, numerous innovations in structure, materials, and operating principles have been introduced. These advancements have significantly reduced the cost, leakage rate, power loss, and dead volume of microvalves while enhancing their response speed and biocompatibility. Microvalves are generally categorized into active and passive types. Passive microvalves utilize their intrinsic structure to achieve valve functionality. For example, the classic Tesla valve leverages a unique asymmetric design that creates direction-dependent resistance within the channel, enabling unidirectional fluid flow (Nguyen et al., 2021) (Figure 3A). Notably, in a seminal article published in *Science* in 2000, Unger et al. (2000) introduced a fabrication method for pneumatic micropumps and microvalves, which has since become a cornerstone in the field. This design, shown in Figure 3B, comprises a three-layer microfluidic structure. The top layer contains gas channels, the bottom layer hosts fluid channels, and an elastic PDMS membrane separates the two. By adjusting the gas pressure in the pneumatic channels, the membrane deflects, allowing the valve to open or close. This approach laid the foundation for subsequent research and development of pneumatic microvalves and micropumps.

#### 2.3.3 Micromixers

Mixers are a critical component of microfluidic chips, enabling thorough and rapid mixing of two or more substances within microfluidic channels. The effectiveness and efficiency of mixing directly influence the overall reaction performance. Based on the presence or absence of external power sources, mixers can be categorized into active and passive types. Active mixers rely on external forces to drive the mixing process, whereas passive mixers achieve mixing by leveraging channel structures to increase the contact area between fluids and guide their interaction.

**FIGURE 4:**
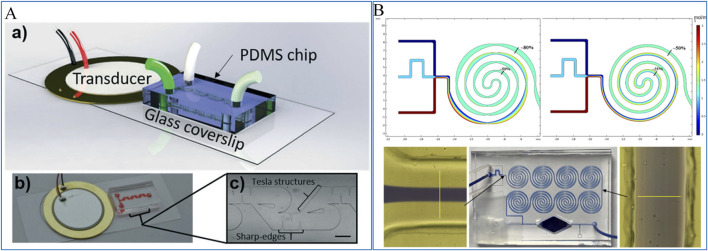
**(A)** A micromixer based on acoustic fluidic control (Bachman et al., 2020); **(B)** A passive micromixer based on divergent convergence and spiral structure (Xue et al., 2021b).

Active mixers utilize external fields, such as thermal, pressure, acoustic, electric, or magnetic fields, to enhance mixing. These methods increase the interaction surface between fluids, disrupt fluid flow, or induce chaotic advection. For instance, as shown in Figure 4A, Bachman et al. (2020) developed an acoustic-field-controlled mixer capable of achieving complete mixing over a wide flow rate range (20–2,000 μL/min). This mixer combines active acoustic streaming for low flow rates with passive hydrodynamic mixing for high flow rates, resulting in excellent mixing efficiency.

In contrast, passive mixers do not require external power sources, making them simpler in design and easier to integrate into microfluidic systems. Passive mixers can be further divided into two-dimensional (2D) and three-dimensional (3D) structures. 2D passive mixers typically use features such as obstacles (Wang et al., 2019), asymmetric convection (Chen et al., 2018), contraction-expansion arrays (Jang et al., 2019), and curved channels (Mehrdel et al., 2018) to induce fluid mixing. Xue et al. (2021b) developed a hybrid passive micromixer by integrating contraction-expansion structures with helical channels. As shown in Figure 4B, simulations were conducted to evaluate the mixer’s performance, showing a 30% improvement in mixing efficiency compared to standard helical passive mixers. This enhancement was further validated through dye-based experiments, confirming the effectiveness of the hybrid design.

## 3 Microfluidic biosensors in rapid detection of foodborne pathogenic bacteria

Microfluidic biosensors integrate the fast and sensitive detection capabilities of traditional biosensors with the unique features of microfluidic systems, such as miniaturization, low reagent consumption, and high integration. These advantages have made them increasingly popular for early screening and rapid detection of foodborne pathogens (Gao et al., 2022; Seidi et al., 2022). Electrochemical and optical biosensors are two widely used types of biosensors, known for their high sensitivity and versatility. This review focuses on summarizing and analyzing the latest advancements in microfluidic electrochemical and optical biosensors, highlighting their applications and potential in the detection of foodborne pathogens. The discussion provides insights into their design principles, operational mechanisms, and innovations that enhance detection capabilities, offering a comprehensive understanding of their contributions to food safety monitoring.

### 3.1 Electrochemical microfluidic biosensors

Electrochemical biosensors can be categorized into various types, including amperometric, impedimetric, potentiometric, and capacitive sensors (Khan et al., 2020). Currently, electrochemical biosensors have been successfully employed in the detection of foodborne pathogens [such as *Salmonella* (Liu et al., 2022; Xue et al., 2021a), *Escherichia coli O157:H7* (Wang et al., 2017), *Listeria monocytogenes* (Lee et al., 2021), and *Vibrio parahaemolyticus* (Jiang et al., 2021)] and food allergens (Su et al., 2021). Numerous studies have demonstrated the significant potential of electrochemical analysis for rapid identification and detection of pathogens (Awang et al., 2021; Castle et al., 2021). Among these, amperometric and impedimetric biosensors are the most widely used for pathogen detection due to their high sensitivity and rapid response. Below we will primarily focus on the application of impedimetric and amperometric biosensors in the detection of foodborne pathogens.

#### 3.1.1 Impedance biosensors

**FIGURE 5:**
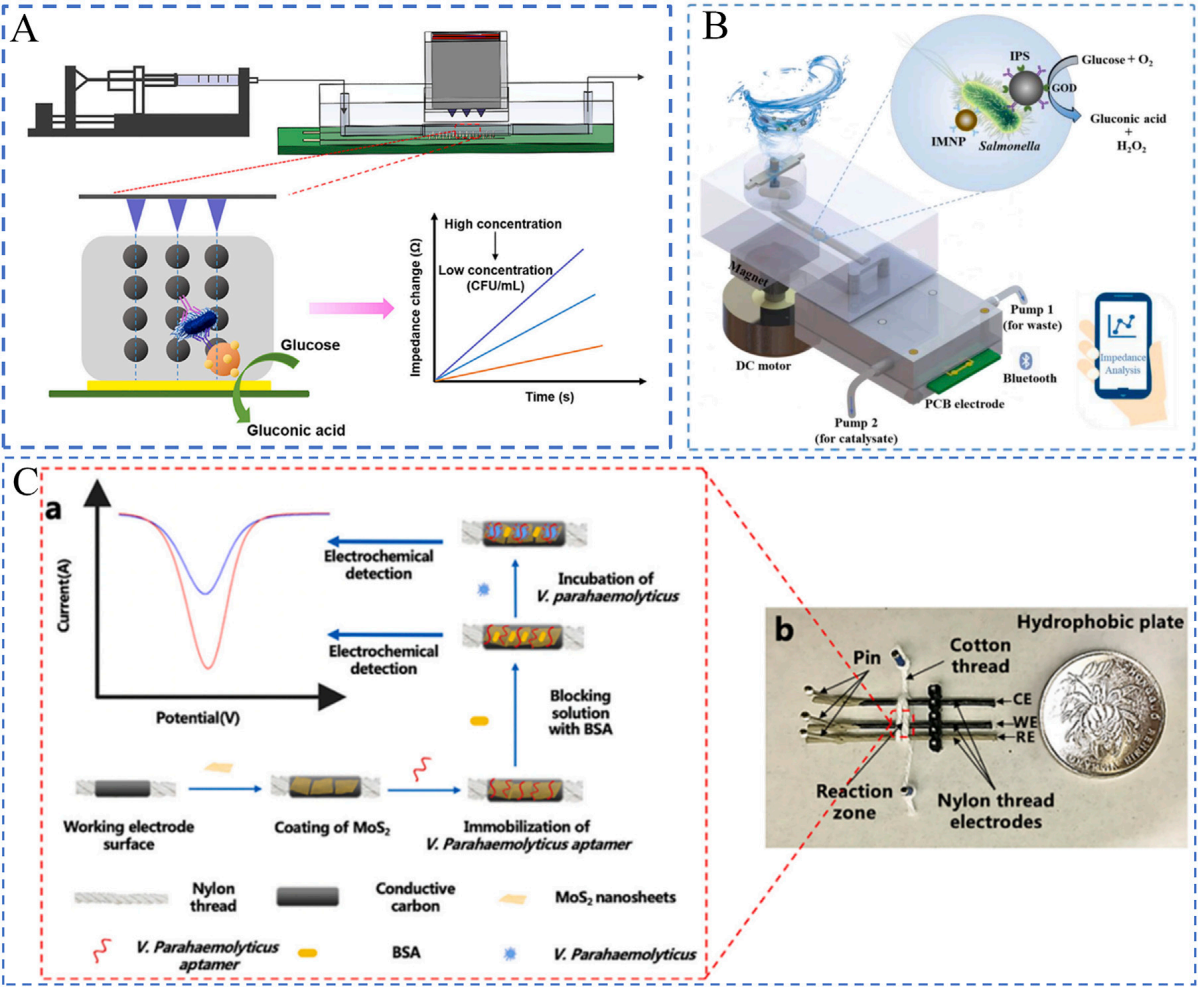
Electrochemical Biosensors. **(A)** Impedance-based biosensor (Jiang et al., 2023); **(B)** Impedance biosensor with interdigitated electrodes (Xue et al., 2023). **(C)** Voltammetric biosensor (Jiang et al., 2021).

The working principle of impedance biosensors involves measuring electrochemical impedance changes at the electrode interface under a constant bias with alternating perturbation. Due to their rapid response, compact design, low cost, and ease of miniaturization, these sensors have been widely applied in the detection of foodborne bacteria. By integrating impedance biosensors with microfluidic channels and microelectrodes, precise and real-time measurements can be achieved. For instance, Jiang et al. (2023) developed a real-time impedance monitoring technique based on magnetic nanobead chains and PCB interdigitated electrodes for the rapid detection of *Salmonella* in food, as illustrated in Figure 5A. First, immunomagnetic nanobeads, target bacteria, and polystyrene microspheres labeled with glucose oxidase are mixed to form a nanobead-bacteria-microsphere complex. These complexes are then injected into a microfluidic chip, where a magnetic grid-enhanced field aligns them into chain-like structures above the electrodes. Subsequently, non-conductive glucose is injected and catalyzed by glucose oxidase to produce conductive gluconic acid and non-conductive hydrogen peroxide. These products rapidly diffuse to the electrode surface, causing impedance changes. Finally, the impedance variations are monitored in real time using PCB interdigitated electrodes to quantify the bacterial concentration. This biosensor demonstrated a good linear response in the range of 1.8 × 10^3^ to 1.8 × 10^6^ CFU/mL, with a detection limit of 50 CFU/mL and a total analysis time of 60 min.

Due to the diversity of different electrodes, Yao et al. (2018) developed a microfluidic impedance biosensor combined magnetic separation and urease catalysis for continuous-flow detection of *E. coli* O157:H7, and demonstrated that impedance normalization was effective to improve sensitivity and reduce the background noise from the variation of different microelectrodes. This study employed magnetic nanoparticles (MNPs) modified with antibody and gold nanoparticles (GNPs) modified with the urease and the aptamers to capture and label with the bacteria to form the MNP-*E. coli*-GNP-urease complexes. The complexes were used to catalyze the hydrolysis of urea into ammonium carbonate, leading to the decrease in the impedance. The impedance was online measured by this microfluidic electrochemical sensor and analyzed using the impedance normalization to determine the concentration of *E. coli* O157:H7. This biosensor had a good linear relationship between the relative change rate of impedance and the concentration of *E. coli* O157:H7 from 10^1^ to 10^5^ CFU/mL and the LOD was as low as 1.2 × 10^1^ CFU/mL within 2 h of the overall time for detection (including sample incubation and impedance measurement).

To further enhance detection performance, Xue et al. (2023) developed microfluidic platforms for the rapid and automated detection of *Salmonella*, as illustrated in Figure 5B. This platform integrates technologies such as continuous-flow magnetic separation, enzymatic impedance amplification, and smartphone-based data analysis. The detection process begins by simultaneously introducing magnetic nanoparticles (MNPs) conjugated with polyclonal antibodies, polystyrene spheres (PSs) functionalized with monoclonal antibodies and glucose oxidase, and bacterial samples into a reaction chamber. These components are thoroughly mixed using an active stirrer, forming MNP-*Salmonella*-PS sandwich complexes. The complexes are then captured in the separation channel using an enhanced magnetic field and washed with deionized water to remove excess immuno-PSs and residual ions. Finally, the catalytic products are dynamically measured via impedance changes using PCB electrodes integrated into the chip, while a custom-developed smartphone application analyzes the data to quantify the bacterial concentration. The sensor demonstrated the capability to quantitatively detect *Salmonella* in the range of 1.3 × 10^2^ to 1.3 × 10^6^ CFU/mL within 1.5 h, with a detection limit of 53 CFU/mL.

Liu et al. (2022) introduced a microfluidic biosensor based on magnetic separation, enzymatic catalysis, and electrochemical impedance analysis for the rapid detection of *Salmonella*. In this system, bacterial samples, magnetic nanoparticles (MNPs) modified with capture antibodies, and enzyme probes functionalized with detection antibodies and glucose oxidase (GOx) were simultaneously injected into the microfluidic chip. Following mixing and incubation, MNP-bacteria-probe complexes were formed. Subsequently, high-impedance glucose was introduced into the chip and catalyzed by GOx on the complexes, converting it into high-impedance hydrogen peroxide and low-impedance gluconic acid. Finally, the products were quantified using low-cost interdigitated microelectrodes and an electrochemical impedance analyzer, allowing the determination of bacterial concentration. The results demonstrated that this biosensor could quantitatively detect *Salmonella* concentrations ranging from 1.6 × 10^2^ to 1.6 × 10^6^ CFU/mL within 1 h under optimal conditions, with a detection limit of 73 CFU/mL. Furthermore, the biosensor exhibited excellent feasibility in real-world applications, successfully detecting *Salmonella* spiked in chicken samples.

To further enhance detection accuracy, Karuppiah et al. (2021) developed an ultra-sensitive, low-cost paper-based graphene oxide nanobiosensor for monitoring bacterial contamination in water. The method involves using screen-printing technology to deposit graphene ink onto hydrophobic paper, followed by the deposition of graphene oxide (GO) on the graphene surface. Subsequently, the biological recognition element Concanavalin A (ConA) is covalently bonded to the GO surface, forming the GGO_ConA biosensing electrode. The resulting biosensor exhibits rapid electron transfer capability and a large electrochemical active surface area. Finally, the biosensor’s performance was evaluated in sludge water and beach water using electrochemical impedance spectroscopy (EIS). The results show that the charge transfer resistance (Rct) of the GGO_ConA electrode increases linearly with bacterial concentration, achieving a limit of detection (LOD) of 10 CFU/mL, which is 100 times more sensitive than previously reported methods. Potential solutions to improve the stability of industrialized impedance biosensors include better control of electrode fabrication during large-scale production, as well as self-calibration during testing to account for electrode variations, ensuring the repeatability of the results.

#### 3.1.2 Amperometric biosensors

Amperometric biosensors, also known as current-based biosensors, are novel devices that generate and measure current signals based on electrochemical oxidation or reduction reactions. These biosensors primarily rely on cyclic voltammetry (CV), where the electrode potential is controlled to alternately drive redox reactions on the electrode surface. By analyzing the current-potential curves, the target analyte on the electrode surface can be quantitatively determined. Amperometric biosensors based on CV, when combined with immunological techniques, have been widely applied for the sensitive detection of foodborne pathogens. For instance, Jiang et al. (2021) proposed a thread-based microfluidic electrochemical aptasensor for the rapid detection of *Vibrio parahaemolyticus* in seafood, as shown in Figure 5C. The sensor utilizes threads as the substrate material, with microfluidic channels constructed directly on the threads. The microfluidic electrode channels are formed by wrapping cotton threads around the three-electrode system, while molybdenum disulfide (MoS₂) nanosheets are incorporated to enhance the sensitivity of electrochemical measurements. When the target bacteria (*Vibrio parahaemolyticus*) bind to the aptamer on the electrode surface, the resulting charge changes at the electrode interface are detected via electrochemical methods such as CV. The sensor demonstrates a dynamic detection range of 10 to 10^6^ CFU/mL with a detection limit of 5.74 CFU/mL. Compared to conventional plate counting methods, this sensor offers higher sensitivity, shorter detection times, and maintains high specificity and accuracy.

To further enhance detection sensitivity, Singh et al. (2018) developed a sensitive and selective microfluidic immunochip for detecting *Salmonella typhimurium* cells. The sensor employs a composite material of carboxylated multi-walled carbon nanotubes (cMWCNTs) wrapped with graphene oxide (GO) nanosheets as the sensing material. The colloidal solution of the GO-cMWCNTs composite was selectively deposited onto a patterned indium tin oxide (ITO) electrode and sealed with polydimethylsiloxane (PDMS) microchannels. Covalent biofunctionalization of the *S. typhimurium* antibody (StAb) was performed *in situ* on the composite material using EDC-NHS chemistry. Results show that wrapping cMWCNTs with GO significantly improved electron transfer behavior, nearly doubling the sensitivity to 162.47 µA/CFU^−1^/mL·cm^−2^, compared to 89.16 µA/CFU^−1^/mL·cm^−2^ for sensors using GO sheets alone. Das et al. (2019) proposed a method for rapid, accurate, and on-site detection of *Pseudomonas aeruginosa* (PA) using the peroxidase-mimicking activity of gold nanozymes (NanoZyme) in combination with aptamer technology. The sensor employs a PA-specific aptamer (F23) adsorbed onto the surface of gold nanoparticles (GNPs), inhibiting their peroxidase-mimicking activity. In the presence of PA, the aptamer binds to PA and detaches from the GNPs, restoring the NanoZyme’s peroxidase-mimicking activity. This activity catalyzes the oxidation of 3,3′,5,5′-tetramethylbenzidine (TMB), producing a blue-colored product that allows colorimetric detection by the naked eye. Finally, the oxidized TMB solution is deposited onto a commercial carbon screen-printed electrode, and the electrochemical signal is measured amperometrically for high-sensitivity detection. Results indicate that the presence of PA significantly enhances the electrochemical signal, achieving a detection limit as low as 60 CFU/mL with a detection time of 10 min. The amperometric analysis is highly sensitive for the detection of pathogenic bacteria. However, most current-type biosensors rely on cumbersome labeling to enhance the electrochemical reactions on the electrode surface, which limits their field application.

### 3.2 Optical microfluidic biosensors

Optical biosensors, with their non-contact detection characteristics, have been increasingly integrated into microfluidic chips. The tight integration of optical detection systems with microfluidic chips enhances the functionality and performance of these devices, leading to the development of unique, compact structures that improve detection accuracy and resistance to interference. As a result, microfluidic optical biosensors have become a research hotspot in pathogen detection. In this section, we discuss the applications of microfluidic technology combined with surface plasmon resonance (SPR), fluorescence, and colorimetric sensors in the detection of foodborne pathogens.

#### 3.2.1 Surface plasmon resonance biosensors

**FIGURE 6:**
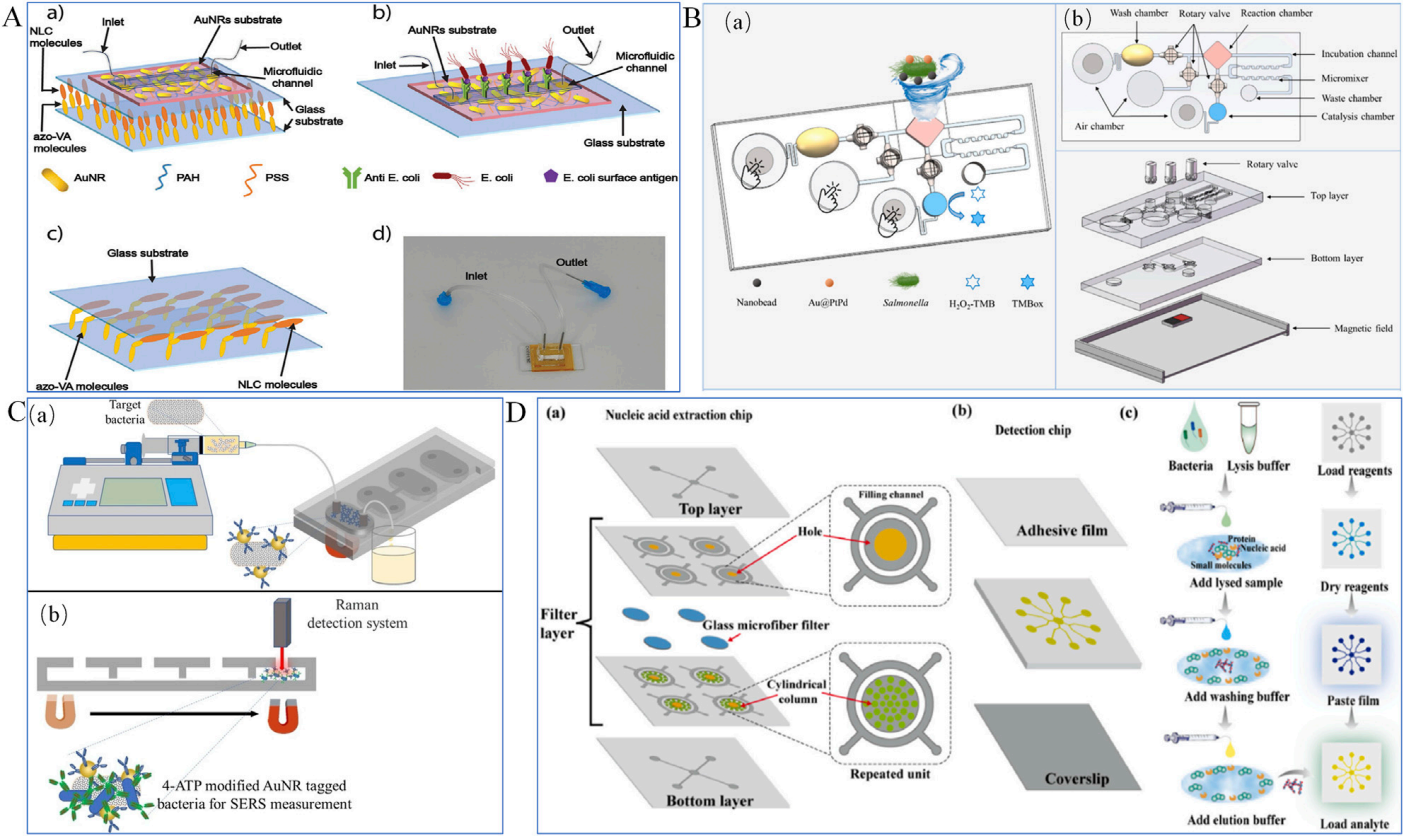
Optical Biosensors. **(A)** Plasmonic biosensor (Sforza et al., 2024); **(B)** Colorimetric biosensor (Jiang et al., 2024); **(C)** Surface-enhanced Raman scattering (SERS) sensor (Dogan et al., 2022); **(D)** Fluorescent biosensor (Shang et al., 2024).

Surface plasmon resonance (SPR) biosensors are optical devices that detect molecular interactions in real-time by measuring changes in refractive index at a sensor surface, caused by the binding of target analytes to recognition elements (Bong et al., 2024). Recent advances have revealed that SPR biosensors can be an efficient tool in detection of foodborne bacteria (Banerjee et al., 2024). For example, to enhance detection accuracy, Soler et al. (2018) developed a portable nanoplasmonic biosensor for rapid and multiplex detection of pathogen infections. This sensor utilizes a nanopore array design, enabling straightforward, high-sensitivity analyses. Integrated with a microarray and microfluidic system, it allows the detection of multiple targets within minutes without requiring sample preprocessing or amplification steps. Results showed that the detection limit of the sensor ranged from 10^2^ to 10^3^ bacteria per milliliter, demonstrating high sensitivity and accuracy. Additionally, Sforza et al. (2024) proposed an innovative cascaded plasmonic-liquid crystal biosensor for rapid detection of harmful bacteria in drinking water, as shown in Figure 6A. This biosensor employs a cascaded structure comprising gold nanorod arrays (AuNRs) and photoresponsive nematic liquid crystals (NLCs). The AuNR arrays, fixed on a glass substrate and integrated with a microfluidic channel, enable direct sampling and analysis of small water volumes, enhancing sensitivity. The sensor detects low concentrations of *Escherichia coli* using localized surface plasmon resonance (LSPR) induced by the AuNR arrays, while NLCs detect high bacterial concentrations through changes in light intensity. The results demonstrated effective detection of low *E. coli* concentrations in the range of 10 to 10^5^ CFU/mL using the AuNR arrays, and high concentrations in the range of 10^6^ to 10^9^ CFU/mL using the NLCs.

To enable real-time detection of pathogens, Xiao et al. (2023) developed a portable smartphone-based imaging surface plasmon resonance (iSPR) biosensor for detecting allergens in plant-based dairy products. The sensor consists of a 3D-printed microfluidic SPR chip and was compared in performance with traditional benchtop SPR instruments. The results demonstrate that the smartphone-based iSPR sensor can detect THP at concentrations as low as 0.625 μg/mL. The limits of detection (LOD) in various diluted plant-based dairy products are as follows: 0.53 μg/mL in soy milk, 0.16 μg/mL in oat milk, 0.14 μg/mL in rice milk, 0.06 μg/mL in coconut milk, and 0.04 μg/mL in almond milk. Most SPR biosensors are still restricted to the laboratory, and their sensitivity for detecting pathogens in food samples should be further enhanced.

#### 3.2.2 Colorimetric biosensors

Colorimetric biosensors are technologies that enable qualitative or quantitative analysis of analytes based on color changes resulting from reactions at different analyte concentrations. With advantages such as non-contact operation, low cost, and easy integration, colorimetric biosensors have been widely used for the rapid detection of foodborne pathogens (Xue et al., 2024). For example, Wang et al. (2021) developed a dual-mode aptamer sensor combining colorimetric analysis and microfluidic chips for simultaneous detection of multiple pathogens in food. The method involves using 4-mercaptophenylboronic acid-modified stirring rods to extract bacteria. After forming a sandwich structure, colorimetric analysis is used to preliminarily determine whether the sample contains target bacteria. Positive samples are further analyzed quantitatively using a microfluidic chip. Experimental results indicate the method performs well in real water samples, with recovery rates between 95.9% and 102% and relative standard deviations (RSD) ranging from 2.7% to 6.2%. This approach is applicable not only to *Vibrio parahaemolyticus* and *Salmonella typhimurium* but also shows potential for extension to other foodborne pathogens. Qi et al. (2022) proposed a power-free microfluidic biosensor for the rapid detection of *Salmonella*. This biosensor integrates a finger-driven micropump, micromixer, and smartphone application to achieve a fully automated process, including sample loading, mixing, incubation, washing, separation, and detection. Gold@platinum nanocatalysts (Au@PtNCs) were employed to amplify signals and enhance detection sensitivity. Experimental results demonstrate that the biosensor can quantitatively detect *Salmonella* in the range of 3.5 × 10^2^ to 3.5 × 10^5^ CFU/mL within 1 h, with a detection limit as low as 350 CFU/mL.

To further improve detection accuracy, Jiang et al. (2024) developed a colorimetric biosensor based on a finger-driven microfluidic chip and gold@platinum-palladium (Au@PtPd) nanocatalysts, featuring rapid, sensitive, and portable characteristics suitable for on-site screening of foodborne pathogens, as illustrated in Figure 6B. The microfluidic chip comprises three air-restricted chambers for precise air control, three rotary valves for selective fluid control, and a convergent-divergent passive micromixer paired with a squeeze-inhale active micromixer for efficient fluid mixing. Immune Au@PtPd nanocatalysts were employed for signal amplification to enhance detection sensitivity. A sliding magnetic field was utilized to capture magnetic nanobead-bacteria-nanocatalyst complexes, enabling target bacteria enrichment and separation. The sensor demonstrated high specificity toward target bacteria, such as *Salmonella typhimurium*, with low responses to non-target bacteria, including *Listeria monocytogenes*, *Vibrio parahaemolyticus*, and *Escherichia coli* O157:H7. It was capable of detecting *Salmonella* at a concentration as low as 45 CFU/mL within 25 min.

Wang et al. (2023) developed a power-free microfluidic biosensor for the rapid detection of foodborne bacteria. This sensor uses manganese dioxide nanoclusters (MnO₂ NCs) as both a fluid driver and signal amplifier, enabling a microfluidic biosensor without the need for external power. Combined with a smartphone app for image processing, the sensor improves detection accuracy and convenience. By testing *Salmonella* in milk and pork samples, the sensor demonstrated the ability to quantitatively detect *Salmonella* at concentrations as low as 63 CFU/mL within 30 min. The recovery rates ranged from 92.77% to 102.05%, indicating its suitability for detecting bacteria in real samples. Man et al. (2023) proposed an integrated distance-based microfluidic aptasensor that utilizes biotin-modified aptamers to react with *Salmonella*. Horseradish peroxidase-labeled streptavidin (HRP-SA) catalyzes hydrogen peroxide to generate oxygen, propelling red gold nanoparticles (GNPs) along a serpentine channel for visual quantitative detection. This sensor integrates sample input and result output, streamlining the process from sample preparation to result interpretation and simplifying operational steps. The results showed that the sensor could detect *Salmonella typhimurium* at concentrations as low as 3.7 × 10^1^ CFU/mL, with excellent reliability, high sensitivity, and specificity. Many studies have indicated that colorimetric biosensors are promising tools for online detection of pathogenic bacteria. However, achieving stable and sensitive colorimetric analysis still requires integration with various technologies, such as smartphone, image processing, and artificial intelligence.

#### 3.2.3 Surface-enhanced Raman scattering (SERS) biosensors

Surface-enhanced Raman scattering (SERS) biosensors are devices that leverage nanostructured metallic surfaces to significantly amplify Raman signals, enabling ultrasensitive and specific molecular detection. Surface-enhanced Raman scattering (SERS) technology, known for its high sensitivity, low detection limits, multiplexing capabilities, and potential in sensor design, has shown significant promise in the fields of bioanalysis and diagnostics. For instance, Dogan et al. (2022) developed a microfluidic chip based on SERS technology for detecting *Escherichia coli* in milk samples, as illustrated in Figure 6C. The detection process begins with immunomagnetic separation (IMS) using antibody-modified magnetic nanoparticles (MNPs) to enrich and isolate the target pathogen. These MNPs bind specifically to *E. coli*, forming MNP-*E. coli* complexes, which are subsequently transferred into the microfluidic chip. In the final microchamber, gold nanorods (Au NRs) labeled with 4-aminothiophenol (4-ATP) are added as SERS probes. These probes interact with the MNP-*E. coli* complexes, forming a sandwich immunocomplex structure. The formation of the sandwich structure induces strong SERS signals from the gold nanorods, enabling quantitative analysis of *E. coli* concentrations. The results indicate that this sensor can detect *E. coli* at concentrations ranging from 10^1^ to 10⁷ CFU/mL, with a total analysis time of less than 60 min. This approach not only improves detection sensitivity and speed but also offers high selectivity and cost-effectiveness, presenting a novel solution for pathogen detection. Although label-free SERS biosensors are less sensitive and more susceptible to interference from complex food matrices, label-based biosensors can detect target bacteria with high sensitivity by reporting the SERS spectra of active labels, it remains a significant challenge to use SERS biosensors as routine tools for detecting pathogenic bacteria in food.

#### 3.2.4 Fluorescent biosensors

Fluorescent biosensors are analytical devices that detect target molecules by generating fluorescence signals through the interaction between biomolecules and fluorescent probes, offering high sensitivity and specificity for real-time monitoring (Liu et al., 2024). The fundamental principle of fluorescent biosensors is to utilize changes in the fluorescence intensity of fluorescent labels bound to the target analyte for qualitative or quantitative analysis. Common fluorescent materials include metal nanoparticles, quantum dots, upconversion materials, and organic fluorescent dyes. Due to their high sensitivity, strong specificity, and non-contact detection capabilities, fluorescent biosensors have become a research hotspot in the field of foodborne pathogen detection (Xue et al., 2020). For example, Shang et al. (2024) designed an integrated microfluidic biosensor (FID-MP) for multiplex detection of foodborne bacteria, aimed at point-of-care testing (POCT) for foodborne pathogens, as shown in Figure 6D. The platform uses a filter membrane for on-chip nucleic acid extraction to obtain bacterial DNA templates. The extracted DNA flows to a detection chip for nucleic acid amplification. The fluorescence images generated during the amplification reaction are read using a smartphone integrated with a portable signal detector. This sensor can detect eight foodborne pathogens with 100% specificity and a sensitivity comparable to standard real-time RPA reactions. The entire process is completed within 60 min, significantly reducing the time required by traditional detection methods.

To further enhance detection sensitivity, Ang et al. (2024) developed a rapid bacterial detection system based on a microfluidic surface acoustic wave-activated nanoseive (SWANS). This system employs a surface acoustic wave (SAW)-driven microfluidic device that uses Bjerknes forces to capture and release bacterial cells. During the capture process, the bacterial cells are fluorescently stained and subsequently detected via fluorescence microscopy. Experimental results demonstrate that the system can detect bacterial concentrations as low as 10 CFU/mL and exhibits high sensitivity to milk samples of varying quality.

To detect multiple pathogens, Feng et al. (2024) designed a microfluidic chip developed using 3D printing technology, integrating an aptamer-based nanointerferometer capable of simultaneously detecting four common foodborne pathogens: *Listeria monocytogenes*, *Escherichia coli*, *Salmonella typhimurium*, and *Staphylococcus aureus*. The chip was designed using AutoCAD software, including components such as droplet inlets, flow channels, check valves, and sensing regions. It was fabricated using a 3D printer, followed by polishing with micro sandpaper and surface modification to improve surface quality. Optical signal changes were monitored using a spectrometer to validate the chip’s detection performance. Results show that the chip reaches saturation within 10–30 min, responds rapidly to low concentrations of foodborne pathogens, and achieves a detection limit as low as 10 CFU/mL, well below the concentrations required to cause illness. Liang et al. (2023) designed a wireless microscopy imaging system combined with an extreme gradient boosting (XGBoost) classification method for detecting bacterial mixtures in water and milk. The system utilizes five quorum-sensing peptides extracted from bacterial biofilms that crosslink with submicron particles. This peptide-bacteria interaction induces particle aggregation on a paper-based microfluidic chip. A wireless fluorescence microscope counts these aggregated particles. The study evaluated four machine learning classifiers, with XGBoost achieving the highest accuracy at 83.75%. Experimental results indicate that the system can identify ten bacterial species, including *Bacillus subtilis*, *Campylobacter jejuni*, and *Enterococcus faecalis*, with sensitivity and accuracy across different bacterial concentrations. The entire detection process is completed within 30 min. Microfluidic fluorescence biosensors have great potential for detection of foodborne pathogens and are expected to be extended to field applications, especially with the development of portable fluorescence detectors. However, their detection performance is always limited by the inherent photophysical drawbacks of fluorophores, such as instability in complex matrices and rapid photobleaching.

### 3.3 Other biosensors

**FIGURE 7:**
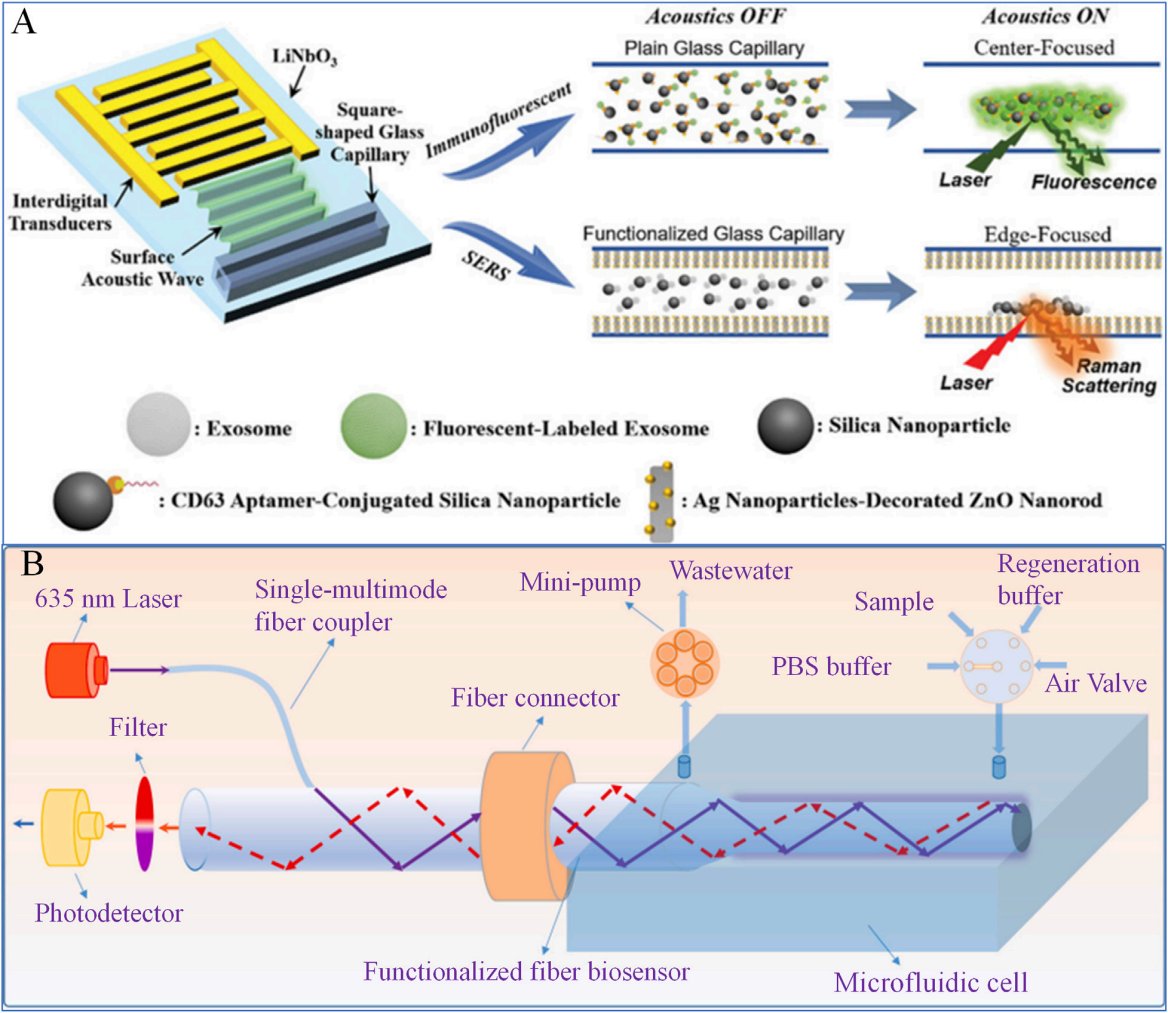
Other Biosensors. **(A)** A dual-mode biosensor platform based on acoustofluidic technology (Hao et al., 2020); **(B)** A microfluidic biosensor for multiplex immunoassays (Han et al., 2024).

Over the past decade, numerous microfluidic biosensors based on diverse technology have been developed for the detection of pathogens, such as magnetic (Chen et al., 2015; Giouroudi and Kokkinis, 2017), acoustic (Nair et al., 2022), thermal (Fu et al., 2021; Guo et al., 2021) and distance-based (Huang et al., 2019; Shao et al., 2019) biosensors. Hao et al. (2020) proposed a dual-modal biosensor platform based on acoustofluidic technology capable of performing both immunofluorescence and surface-enhanced Raman scattering (SERS) detection. This platform utilizes surface acoustic waves to concentrate nanoparticles at the center or edge of a glass capillary, amplifying the detection signals. Specifically, immunofluorescence detection is achieved by focusing fluorescent analytes and functionalized nanoparticles at the microchannel center, while SERS detection is accomplished by concentrating analytes at the microchannel edge, where they interact with a plasmonic ZnO-Ag nanorod array deposited on the inner walls (Figure 7A). The results demonstrated that the dual-modal sensor successfully detected biomarkers, such as exosomes, in real-time at extremely low concentrations (down to tens of exosomes per microliter). The platform shows great potential for foodborne pathogen detection.

Han et al. (2024) introduced a novel sensor called AEFB (Air Exchange-Enhanced Fluorescence Biosensor), designed for rapid and highly sensitive detection of *Escherichia coli* O157:H7. The sensor employs an air-exchange step to significantly enhance the collection efficiency of fluorescence signals, improving detection sensitivity. Additionally, the biosensor integrates CRISPR/Cas12a technology into a 3D-printed microfluidic biochip. Optical fiber biosensors are embedded into the microfluidic chip, enabling sequential injection of buffer solutions, samples, and regeneration solutions through a six-way valve and a miniature pump (Figure 7B). All bacterial strains were cultured overnight in sterile LB broth at 37°C with shaking at 150 rpm, and concentrations were determined using standard plate counting methods. The results demonstrate that the proposed sensor is highly sensitive, accurate, portable, and repeatable for detecting *E. coli* O157:H7. Li et al. (2024) proposed a method to enhance biosensing using acoustofluidics. This sensor incorporates focused traveling surface acoustic waves (FTSAWs) into an acoustofluidic chip, continuously enriching target molecules in a contraction zone for immediate immunoreaction detection, significantly improving sensitivity and speed. Experimental results showed that this method could accumulate large amounts of polystyrene microspheres pre-captured with human IgG within seconds. It achieved rapid detection at concentrations as low as 0.1 ng/mL (∼0.7 pM), offering superior speed and sensitivity compared to conventional methods.

In summary, biosensors have been widely applied in the detection of foodborne pathogens due to their advantages of high sensitivity, ease of operation, and short processing time. However, the characteristics of different biosensors also present certain challenges that require improvement. For instance, in electrochemical biosensors, the output electrical signals and subsequent processing in both current-based and impedance-based biosensors are dependent on large electrochemical workstations, which significantly limits their application in on-site rapid detection. Thus, the miniaturization of electrochemical biosensors holds great promise for expanding their potential applications. While SPR and SERS biosensors enable label-free detection of pathogenic bacteria, the sensitivity of these two biosensors for bacterial detection, especially for label-free strategies, need to be further improved. In the case of fluorescent biosensors, due to the unique properties of fluorescent materials, strict control over certain reaction conditions is required to prevent fluorescence quenching. Therefore, the development of more stable fluorescent materials or optimized fluorescence generation strategies presents a considerable challenge. Colorimetric biosensors can achieve semi-quantitative and qualitative detection without requiring extra equipment, since the produced signal is observed with naked eye or analyzed by a smartphone App. Each type of biosensors has its own advantages and limitations, so it is of great importance to customize an appropriate microfluidic biosensor for a specific application. Additionally, challenges remain in analyzing and handling complex samples owing to the inherent characteristics of microfluidic channels, such as nonspecific adsorption of samples on channel surfaces and channel clogging during complex sample processing. Therefore, it is of significant research importance and value to continuously integrate new technologies and methods into biosensor development to enhance their detection capabilities and performance.

## 4 Recommendations for future research directions

As discussed above, numerous microfluidic biosensors have been studied and shown their broad capacity for fast detection of foodborne bacteria in ensuring food safety. Despite the significant progress, there are still some opportunities to improve the efficiency of microfluidic biosensors for continuous advancement. Below we highlight several future research directions from our perspective, which aim to promote the fast development of this field.

### 4.1 Integrated microfluidic systems

One of the most important future research directions is to develop integrated microfluidic systems. In daily food safety testing, the concentration of pathogenic bacteria in food samples is often extremely low, making it challenging for most microfluidic biosensors to directly and effectively detect these low concentrations of target microorganisms. As a result, separation and enrichment technologies are critical for enhancing sensitivity. Therefore, to achieve specific and sensitive detection, it is necessary to first separate and enrich the target microorganisms. In this way, the development of microfluidic chips or devices that integrate bacterial separation, enrichment, and detection functions can be a major trend for achieving rapid and sensitive detection. A novel microfluidic technology, that enables the integration of separation, enrichment, and sensitive detection on a single chip, can make the process more efficient and user-friendly. By incorporating multiple functional modules, including but not limited to sample preparation, separation, enrichment, and detection, within a microfluidic chip, the detection performance of the whole system can be significantly improved, particularly for these low-concentration targets. Moreover, this integration should substantially reduce detection time, facilitating real-time analysis.

### 4.2 Real-time and on-site detection

It is known that the portability and efficiency of microfluidic biosensors can provide significant advantages for rapid on-site detection. Therefore, the development of real-time and on-site detection should be another future research direction in the field of microfluidic biosensors. In the future, these sensors should enable real-time monitoring throughout various stages of the food supply chain, including production, processing, transportation, and consumption. This capability will allow for the timely identification of harmful substances or pathogenic bacteria in food, helping to prevent contamination incidents. For example, with the use of smartphones or portable devices, both consumers and producers can directly detect issues such as bacterial contamination, heavy metals, and pesticide residues in food, ensuring food safety at every step.

### 4.3 Low-cost and high-throughput detection

Another direction of effort should be put into cost reduction and high-throughput detection. The production and operational costs of microfluidic biosensors are expected to decrease over time, enhancing their potential for large-scale food safety testing. With advancements in technology, the integration level of microfluidic biosensors is expected to continue to improve, enabling future sensors to simultaneously detect multiple food safety parameters on a single platform. For instance, these sensors could concurrently identify a variety of contaminants in food, including bacteria, fungi, viruses, heavy metals, and pesticide residues. This multifunctionality is expected to significantly enhance detection efficiency, reduce time and costs, and meet the high-throughput demands of food safety testing.

### 4.4 Intelligent and automated detection

Last but not least future research direction is to develop intelligent and automated detection microfluidic biosensors to meet future food safety requirements. Microfluidic biosensors can integrate with artificial intelligence (AI) technologies to create intelligent food safety detection systems. AI can assist in analyzing data collected by sensors, enabling automated diagnostics and even predicting potential food safety risks through big data analysis. For example, by incorporating Internet of Things (IoT) technology, these sensors can upload data to the cloud in real-time. The analysis results can be swiftly relayed to relevant personnel, facilitating automated warning systems and decision-support mechanisms.

## 5 Conclusion

This paper reviews and summarizes recent advances in microfluidic biosensors for rapid detection of foodborne bacteria to ensure food safety over the past decade. The findings highlight that microfluidic biosensors exhibit significant potential in food safety monitoring, as they enable rapid and accurate detection of various foodborne pathogens and contaminants. By integrating microfluidic chips with biosensors, sensitivity and specificity can be enhanced, while the required sample volume and analysis time are significantly reduced. Various types of microfluidic-enhanced biosensors have been developed for food safety monitoring, including electrochemical, optical, and other biosensors like biosensors based on acoustofluidic technology. Each type has its own advantages and limitations, with the choice of biosensor depending on the specific application and target analyte.

Overall, microfluidic-enhanced biosensors offer several advantages over traditional methods in food safety monitoring, including portability, low cost, high sensitivity, and rapid analysis time. These features position them as transformative tools in the field of food safety, making the detection and prevention of foodborne diseases easier and faster. However, further research and development are required to optimize the performance of these biosensors and ensure their widespread adoption in the food industry.
